# Chronic gastro-diaphragmatic fistula following sleeve gastrectomy: a diagnostic and therapeutic dilemma

**DOI:** 10.1093/jscr/rjag102

**Published:** 2026-02-26

**Authors:** Caitlin Sorour, Eshwarshanker Jeyarajan

**Affiliations:** Department of General Surgery, Cairns Hospital, 165 The Esplanade, Cairns, QLD 4870, Australia; Department of General Surgery, Cairns Hospital, 165 The Esplanade, Cairns, QLD 4870, Australia

**Keywords:** laparoscopic sleeve gastrectomy, gastric sleeve, gastric leak, gastric fistula, leak

## Abstract

Laparoscopic sleeve gastrectomy (LSG) is an effective bariatric procedure; however, gastric leaks remain a serious complication and are often diagnostically challenging. We describe a case of a chronic gastro-diaphragmatic fistula diagnosed 11 months following LSG. A 46-year-old female initially developed a contained gastric leak four weeks post LSG, managed with endoscopic primary closure. Despite reassuring imaging and endoscopic findings, she experienced persistent dysphagia, post-prandial vomiting, and referred left shoulder pain. Nearly one year after surgery, the presumed culprit of mediastinal migration of the proximal stomach was repaired laparoscopically; however, a small left crural collection was found without identifiable leak. Post-operatively, she deteriorated from an uncontained gastric leak, requiring multiple surgical interventions and prolonged critical care. This case highlights the limitations of imaging studies in detecting chronic, contained leaks and underscores the need for a high index of suspicion in patients with persistent, unexplained symptoms following LSG.

## Introduction

A laparoscopic sleeve gastrectomy (LSG) is an effective weight loss procedure that has become widely practiced [1, 2]. While there are significant benefits to a LSG including gastrointestinal continuity and the ability to convert to other bariatric procedures, it still carries risk of significant complications with one of the most challenging being gastric staple line leaks [3]. The diagnosis of acute and chronic gastric leaks can be a diagnostic and therapeutic challenge with no standardized protocols established due to their varied presentations and management complexities [3, 4]. This case describes an unusual presentation of a chronic gastric leak diagnosed 11 months post LSG leading to a gastro-diaphragmatic fistula.

## Case report

**Figure 1:**
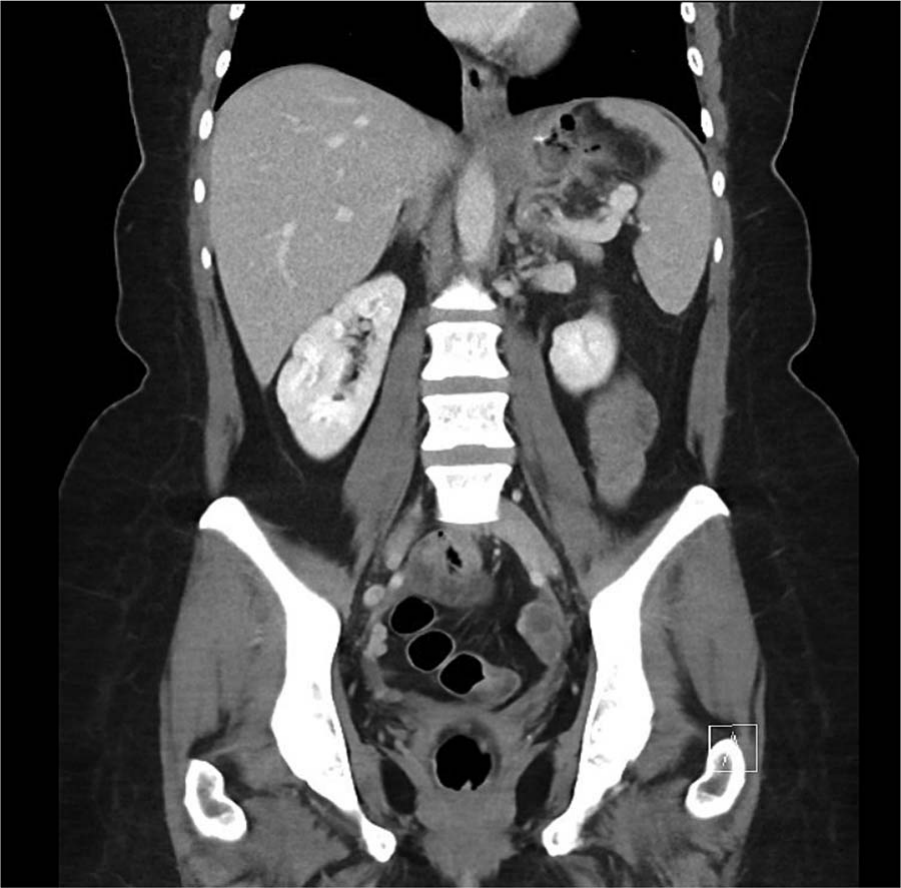
Coronal portal venous phase CT scan of the abdomen and pelvis demonstrating the proximal gastric leak with extra-luminal air in the gastrosplenic ligament.

A 46-year-old female underwent an uncomplicated LSG for weight loss. Four weeks post-operatively she represented with epigastric pain and was diagnosed with an acute, contained leak on computed tomography (CT) (Fig. 1). The patient at the time was systemically well with a normal white cell count (WCC) and a C-reactive protein (CRP) of 67. Given this, a conservative approach was proposed with endoscopic internal drainage using double pigtail stents in conjunction with nasojejunal (NJ) feeds, and/or endoscopic negative pressure vacuum therapy. However, the patient was not willing to pursue enteral feeding or internal drainage and ultimately opted for primary closure of the defect endoscopically which was performed using argon beam coagulation and two X-tack suture devices.

**Figure 2:**
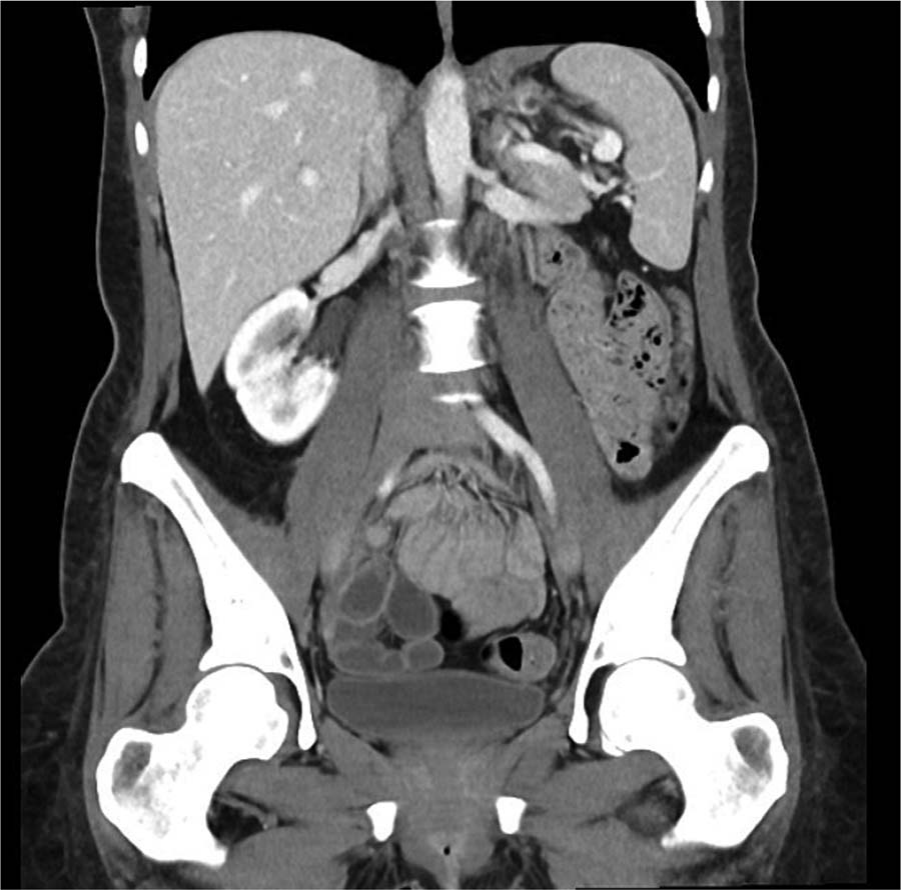
Coronal portal venous phase CT scan of the abdomen and pelvis demonstrating the thickening of the left hemidiaphragm but nil evidence of ongoing leak.

On follow up six months post-procedure, a CT scan demonstrated no ongoing collection with mild thickening of the left hemidiaphragm (Fig. 2), and a barium swallow showed normal passage of contrast. While radiographically mostly reassuring, clinically the patient reported persistent dysphagia and post-prandial vomiting resulting in significantly reduced oral intake and weight loss. Notably, she also reported left shoulder pain associated with eating. A gastroscopy was performed wherein mediastinal migration of the proximal staple line was diagnosed. The previous defect located at the proximal staple line of the stomach remanent was interrogated but the repair remained intact.

Given there was no radiographic or endoscopic evidence of a persistent leak or collection; the leading differential for the cause of the patient’s obstructive symptoms was migration of the staple line above the diaphragm through a narrow crural aperture. Given the presumed diagnosis, patient’s unsustainable weight loss and worsening micronutrient deficiencies, the patient proceeded to a laparoscopic hiatal hernia repair, now almost one year post LSG. Intra-operatively, a small type one hiatal hernia was diagnosed; however, an unusual finding of a small collection located at the left diaphragmatic crus was found and debrided. An on-table gastroscopy and methylene blue leak test were performed but no leak could be identified.

**Figure 3:**
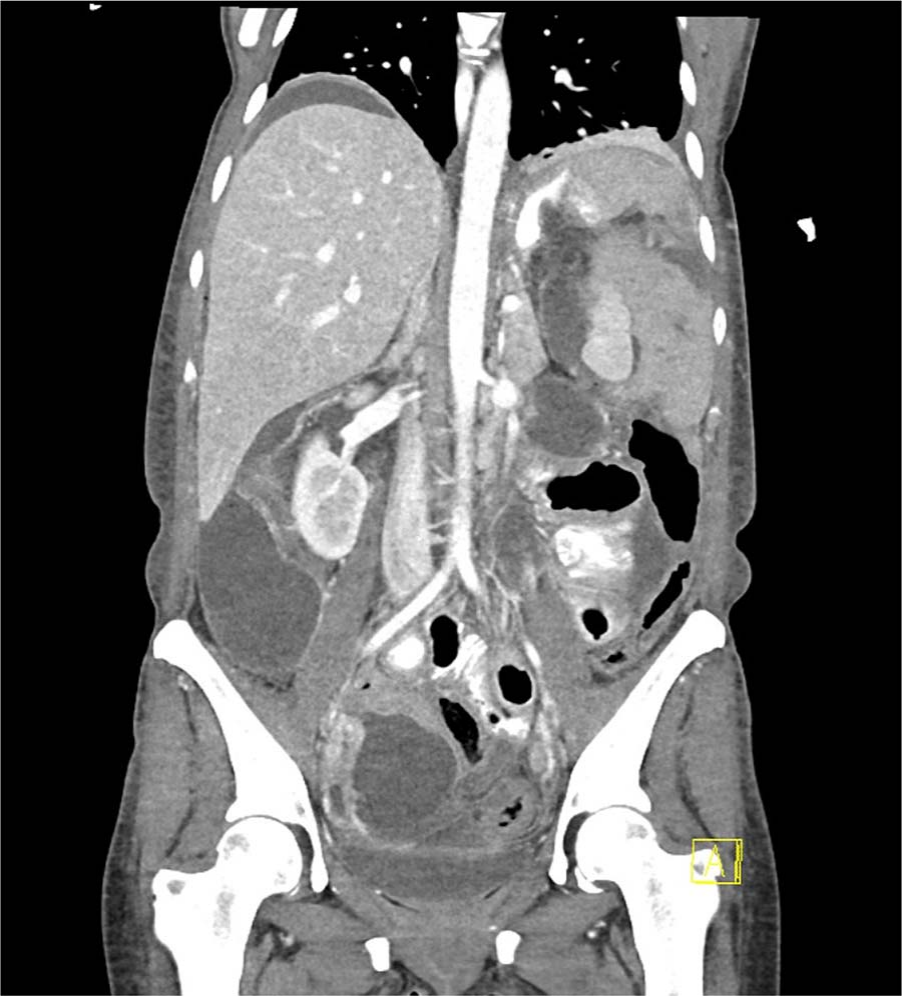
Coronal portal venous phase CT scan with oral contrast of the abdomen and pelvis demonstrating the proximal gastric leak with large volume contrast extravasation into the left upper quadrant and widespread intra-abdominal fluid with pneumoperitoneum.

Unfortunately, day six post-operatively she represented with significant abdominal pain with a WCC of 12.5 and a CRP of 527. A CT demonstrated a large leak from the proximal stomach with widespread fluid and pneumoperitoneum throughout the abdomen (Fig. 3). She proceeded to an urgent diagnostic laparoscopy which found four quadrant peritonitis with food matter and purulent collections throughout the abdomen. A washout was performed laparoscopically though the defect was not able to be identified, and large bore drains were placed at the presumed leak site at the proximal stomach with the aim to transfer to a quaternary center for ongoing care under a specialized unit experienced in management of complex bariatric complications.

While initially the patient recovered well, regrettably three days later she once more developed worsening abdominal pain with frank peritonism and a repeat CT scan showing a persistent, worsening leak with significant intra-peritoneal fluid. She underwent an emergent laparotomy which found gross contamination of the abdomen with an 8 mm defect on the left lateral side of the gastro-oesophageal junction (GOJ). An extensive washout and on-table gastroscopy were performed inserting both NJ tube and a wide-bore gastrostomy tube through the defect. There was concern for colonic distension and caecal ischemia in the setting of worsening septic hypotension and therefore the patient was transported to the intensive care unit with an open abdomen. She returned two days later for a re-look laparotomy and closure with no further significant findings. She was placed on broad spectrum antimicrobials including fluconazole and piperacillin/tazobactam with early total parenteral nutrition and enteral feeding via the NJ. She recovered slowly over a prolonged admission with progressive removal of drains and eventual trial of oral feeds leading to removal of her NJ. The gastrostomy remains, with the aim to progressively downsize the tube via endoscopic guidance to create a controlled gastrocutaneous fistula.

## Discussion

This case outlines an unusual presentation of a chronic gastric leak post LSG and highlights the pitfalls of both diagnosing and managing this rare complication. The incidence of gastric leaks in LSG is estimated to be around 1% to 3% and typically occurs at the left proximal stomach just below the GOJ [5–7]. While the majority of leaks present within the first six weeks post LSG, chronic leaks occurring beyond twelve weeks do occur rarely and have been associated with increased morbidity and prolonged recovery [3, 5, 8, 9].

Clinical manifestations of gastric leak are highly variable with the broad symptoms of tachycardia and fever being the most common [3, 10]. Although CT imaging is regarded as the diagnostic modality of choice, this case demonstrates sensitivity may be limited in the setting of a well-contained fistula [10]. Similarly, endoscopic evaluation may fail to identify small or intermittently draining defects as evidenced by the negative leak test performed intra-operatively in this case.

Management of proximal leaks is difficult and varies significantly on patient factors and diagnostic timing. Endoscopic management with stenting, vacuum therapy or internal drains is generally favored for earlier leaks, while revisional surgery is often required for chronic leaks [3, 4, 11]. In this patient, it is postulated that initial endoscopic closure resulted in the development of a chronic, contained gastro-diaphragmatic fistula, which subsequently became uncontained following surgical debridement, precipitating fulminant peritonitis. This case highlights the limitations of current diagnostic tools and underscores the need for a high index of suspicion in patients with persistent, unexplained symptoms following LSG.
